# The effects of social support and self-efficacy on hopefulness in low-income older adults during COVID-19 pandemic

**DOI:** 10.1186/s12877-024-04915-4

**Published:** 2024-04-02

**Authors:** Soonhyung Kwon, Ellen Benoit, Liliane Windsor

**Affiliations:** 1 Institute for Health, Health Care Policy and Aging Research, Rutgers, the State University of New Jersey, 112 Paterson St, New Brunswick, NJ 08901 USA; 2 North Jersey Community Research Initiative, 393 Central Ave, Newark, NJ 07103 USA; 3 School of Social Work, University of Illinois at Urbana-Champaign, 1010 W Nevada St, Urbana, IL 61801 USA

**Keywords:** Social support, Self-efficacy, Hopefulness, Mediation analysis

## Abstract

**Background:**

Social support and self-efficacy play a significant role in improving positive psychological well-being in marginalized older adults. However, to date, there are few studies identifying the relationships during the COVID-19 pandemic. We examined the effect of social support and self-efficacy on hopefulness in a majority Black sample of marginalized low-income older adults during the COVID-19 pandemic.

**Methods:**

This study used baseline data from a clinical trial designed to increase COVID-19 testing in Essex County, NJ, United States. The dataset involved participants 50 years old or older. We conducted: 1) cross-sectional descriptive/frequency statistics to understand the sociodemographic characteristics, 2) multivariate linear regression to investigate the direct relationships between social support subscales or self-efficacy and hopefulness, and 3) mediation analyses to examine the mediating role of self-efficacy in the relationship between social support and hopefulness.

**Results:**

Our findings showed that self-efficacy had a partial mediating effect on the relationship between social support and hopefulness. After adjusting for covariate variables, social support subscales (i.e., emotional/informational, tangible, affectionate, positive social interaction social support) and self-efficacy were significantly associated with hopefulness. The indirect effect of social support via self-efficacy was positive and statistically significant.

**Conclusion:**

Self-efficacy mediated the relationship between social support and hopefulness in marginalized older adults aged 50 and over. Further research is needed to identify the various facets of positive psychological well-being using longitudinal data and a larger sample size.

Guidance on social distancing designed to mitigate the harm caused by the Coronavirus disease 2019 (COVID-19) increased isolation among older adults by blocking access to social services and social support [1–3]. Isolation during the pandemic increased negative emotions (e.g., fear, anxiety, and stress) and reduced positive emotions (e.g., hopefulness) in people of all ages [4, 5]. A previous study indicated that older age, fewer social activities, psychological distress, and cognitive impairment were likely to decrease hopefulness in older adults [6]. Moreover, a lack of hopefulness was associated with high depressive symptoms and suicidal thoughts [7]. On the other hand, increased positive emotions can predict a lower risk of mortality [3]. Especially during the pandemic, increased negative emotions in older adults were significantly related to a high risk of mortality due to medical disorders or suicide [8].

Hopefulness is one of the most important facets of positive psychological well-being [9]. Hope is “goal-directed thinking in which a person has the perceived capacity to produce routes to desired goals (pathways thinking), along with the motivation to initiate and sustain the use of those routes (agency thinking)” (p. 217) [10]. Older adults with high hopeful thoughts show a higher chance of reaching goals and are closer to their goals than those with low hopeful thoughts [10]. As the future-oriented facet, hopefulness can help older adults shape their optimistic thoughts during the pandemic [11] and correlate with other positive emotions (i.e., gratitude and optimism) [12] in order to avoid negative emotions.

According to the Broaden-and-Build Theory, positive emotions (i.e., hopefulness, joy, interest, contentment, pride, and love) help promote upward spirals toward enhanced psychological or emotional well-being [13] and have longer-lasting effects than negative emotions [14]. These positive emotions encourage new ways of thinking, trying new activities, and the formation of relationships. As a result, they build enduring personal resources (e.g., social support, resilience, skills, and knowledge) [13]. Such an upward spiral enhances physical health, survival, and feelings of fulfillment in life, which increases experiences of positive emotions [13]. As part of the explanation, it is important to identify the relationship between social support and hopefulness among marginalized older adults during the pandemic.

Black adults are less likely than their White peers to report mental illness [15] or substance use disorders and hopelessness [16], but those conditions may be underdiagnosed because of a lack of culturally appropriate screening tools and inequitable access to care and because symptoms of those conditions are more likely to be treated as criminal behavior among people of color [17]. Between 2010 and 2020, rates of suicide increased by 43% among Black Americans, compared with 12% among Whites [17]. Among older Black Americans, chronic racial discrimination was associated with higher odds of psychiatric disorders and psychological distress [15]. Research has found that cumulative routine discrimination increased hopelessness among older Black adults but that social support had some protective effects [18]. Black Americans also experience disproportionately low life expectancy (72 years of life for Blacks compared to 86 years for Asians) [19]; thus, reaching 50 years of age among Black Americans can signify a stage in the life course from midlife to older adulthood.

Older Black adults were more likely than their White peers to live in multi-generational households [20], which might increase access to social support during pandemic conditions but also increases the risk of infection [21, 22]. One study of older Black Americans found that emotional support from family members was not protective for psychiatric disorders but that negative interactions with family members were either a strong risk factor for mental illness or a consequence thereof [23]. Black Americans were less likely than Whites to have home broadband service (71% vs. 80%) [24]. Therefore, coping with isolation during sheltering-in-place requirements might be more difficult for older Black Americans, especially those who live alone [21].

Despite indicating the potential benefits of hopefulness in older adults, most of the studies currently available have significant limitations. Previous studies during the early pandemic captured unique experiences of adverse mental health, such as depression and anxiety, instead of positive psychological well-being, such as hopefulness. Additionally, there is a lack of studies targeting Black adults in their samples and disaggregation of results by race. Given the disproportionate burden of COVID-19 prevalence, death, and complications experienced by socially and medically vulnerable individuals in the United States, it is critical to better understand the role of social support and self-efficacy on hopefulness as potential protective factors in this population.

## Social support

Social support generally plays an essential role in ameliorating adverse mental health [25] and improving hopefulness in older adults [26, 27]. Social support is conceptualized as interpersonal relationships through: “The provision of emotional, informational, or instrumental resources and influencing cognition, emotions, and actions without explicitly aiming to help or support” (p. 102) [28]. Given the narrowed social networks relevant to social support in older adults due to retirement [29] and shelter-in-place policies [1–3], some received social support from their family members more than others (i.e., friends or outside society) during the pandemic [30]. Others maintained social connections virtually and did not demonstrate negative psychosocial consequences [31]. Some COVID-19 research has found that older adults reported isolation as a major source of stress but social connections with family and friends as their greatest source of comfort [32] and that face-to-face (but not digital) contact with social networks significantly mitigated mental health challenges [33]. Social support from family and friends is likely to increase the motivation to achieve hopeful thoughts [10, 34, 35].

## Self-efficacy

Self-efficacy is an individual’s belief (confidence) in the ability to influence the events that affect their lives [36]. Self-efficacy positively predicts an increase in older adults’ positive emotions, such as optimism [37] and happiness [38]. Moreover, self-efficacy works as a mediator in the relationship between social support and subjective well-being [39], and mental health [40]. Accordingly, older adults with high levels of self-efficacy are more likely to cultivate higher positive emotions when compared to those with low levels of self-efficacy. During the pandemic, self-efficacy was strongly associated with older adults’ intentions to stay home in a predominantly (77.3%) White sample [41]. Other research, with a more diverse sample, found increased self-efficacy among older adults aging in place in their homes during the first six months of the pandemic [42].

Despite the large and multidisciplinary body of research on the relationship among social support, self-efficacy, and positive emotions in older adults, to our knowledge, they have not focused on hopefulness in older Black adults in marginalized communities. Adverse mental health (e.g., depression) has been the primary outcome in older adults during the pandemic [43]. With this concern in mind, our study aims to identify the effect of social support and self-efficacy on hopefulness in a sample of majority older Black adults with low-income during the COVID-19 pandemic.

## Methods

### Study participants

A cross-sectional baseline sample of all participants who were 50 or older (*n* = 222), selected from an existing dataset of 676 people who were enrolled in a clinical trial designed to increase COVID-19 testing (3R01MD010629-04S2; PIs: Liliane Windsor and Ellen Benoit), were included in the current analysis. Study data was collected between 2021 and 2023. Eligible participants were: 1) 50 years or older, 2) residents of Essex County, NJ, 3) at high risk of contracting SARs-CoV-2 or developing COVID-19-related death and complications, 4) able to speak English, and 5) willing to provide informed consent.

### Community characteristics

Essex County is a historically marginalized county with a complicated history of struggle based on racism and exclusion [44]. It currently has a population of 800,000, with a high cost of living and a low per capita income of $42,028. The poverty rate in Essex County (15%) is higher than the national average. The largest racial/ethnic group in the population is Black (42%), followed by White (30%), Latinx (24%), and Asian (6%) [45]. Essex County is among the lowest-ranked counties for health in NJ, and 14% of the population is over the age of 65 [45]. The county’s premature death rate is 7,900 compared to 6,300 in NJ. Between 2018 and 2020, the leading causes of death included cancer, heart disease, accidents, COVID-19, and diabetes. Essex County had some of the worst COVID-19 outcomes in NJ, with a total of 277,778 cases and 464 deaths per 100,000 residents in 2022 [46].

Community partners were involved in all aspects of the parent study and the conceptualization and interpretation of findings in the current paper. Authors presented the paper’s findings to the study’s community collaborative board who helped interpret and conceptualize the findings. They understood the need to begin with a cross-sectional analysis in this paper, but encouraged continuing the research with longitudinal data, to capture whether and how hopefulness may change over time, particularly during the pandemic. They also suggested future analyses focused on how hopefulness relates specifically to older adults’ attitudes and intentions toward COVID testing and vaccination.

### Participant recruitment and data collection

Informed by community-based participatory research (CBPR) principles, a well-established community collaborative board (CCB) disseminated the study to the community. The CCB and full-time trained outreach workers followed social distancing guidelines and posted fliers at bus stops, health care agencies, churches, bulletin boards, grocery stores, pharmacies, and social service agencies. People interested in participating called the study’s cell phone number and completed a brief phone screening with outreach workers. Eligible participants were invited to provide informed consent and complete the baseline survey online via Redcap. Participants received $40 cash to complete the 60-minute online survey. This study was approved by the Institutional Review Board from the North Jersey Community Research Initiative (IRB FWA# 00001870).

### Measures

#### Hopefulness

We asked a single question to investigate participants’ hopefulness: *In the past 7 days, how often have you felt hopeful about the future?*, which was adopted from the COVID-19 and Mental Health Measurement working group at Bloomberg School of Public Health, Johns Hopkins University [47]. This measurement was developed to understand how the COVID-19 pandemic affects people’s emotional and mental health. The question measured hopefulness with a 4-point Likert response scale (1 = not at all or less than 1 day, 2 = 1 - 2 days, 3 = 3 - 4 days, and 4 = 5 - 7 days). Higher scores signify higher levels of hopefulness.

#### Self-efficacy

Self-efficacy was investigated by the General Self-Efficacy Scale (GSE). The GSE is a 10-item psychometric scale that asks about optimistic self-beliefs to manage various challenges in life [48]. The score of each item in the GSE ranges from 1 (not at all true) to 4 (exactly true) and was calculated by the average of all items. The GSE showed good internal consistency reliability between 0.76 and 0.90 (Cronbach’s alpha) [48]. Higher scores signify higher levels of self-efficacy.

#### Social support

Social support was measured by the MOS Social Support Survey (MOS-SSS), which is widely used to assess social support [49]. This self-administered social support survey was designed to measure various dimensions of social support [49]. The MOS-SSS consists of 8 emotional/informational support items, 4 tangible social support items, 3 affectionate social support items, 3 positive social interaction social support items, and 1 affectionate support. To obtain a score for each subscale, we averaged the scores for each item in the subscale. The total score was calculated by the average of all items for mediation analysis. The total score ranges from 1 (none of the time) to 5 (all the time). The MOS-SSS has excellent internal consistency reliability (Cronbach’s alpha = 0.97) [49]. Higher scores signify higher levels of social support.

#### Covariate

Covariate variables include age (continuous), marital status (yes, no), gender (male, female), education levels (have never gone to school, grades 5^th^ or less, 9^th^ to 12^th^ grade, no diploma, high school graduate, or General Educational Development (GED) completed some college level or technical or vocational degree, Bachelor’s degree, and other advanced degrees - Masters and doctoral degree), race (Caucasian American, African American, and other – Asian, Native American, and Latin/Hispanic American), income (less than $15,000, $15,000 - $19,999, $20,000 - $24,999, $25,000 - $34,999, $35,000 - $49,999, $50,000 - $74,999, and $75,000 - $99,999), and subjective health (poor, fair, good, very good, and excellent).

### Analysis

Our analysts performed cross-sectional descriptive/frequency statistics, multivariate linear regression, and mediation analysis. Covariate variables such as age, marital status, gender, education levels, race, and subjective health were controlled for in the multivariate regression and mediation analysis. However, income level was excluded from the analysis because the majority of our participants were low-income. The results are presented as unstandardized coefficients (b) and standard error (se), with positive coefficients indicating higher hopefulness levels. Regarding mediation analysis, we conducted 5,000 bootstrap resampling techniques and generated 95% confidence intervals to identify the direct and indirect effects using the PROCESS macro version 4.2 for SPSS [50]. We employed listwise deletion to deal with missing values. All data analyses were performed in SPSS version 20.

## Results

Table 1 indicates the demographic characteristics of participants. The average age of the participants in this study was 58 years old. Ninety-one participants were female, and 198 were married or living with a partner. A majority of participants completed high school or obtained a GED. Three-fourths of the participants were Black. Most participants’ annual incomes were below $50,000. The average subjective health score was rated as lower than “good”. The average score of self-efficacy was 3.05 points, social support subscales were higher than “some of the time”, and hopefulness was 2.21 points.

**Table 1:** Demographic characteristics (*n* = 222)

Variable	
Age, M (SD)	58 (5.92)
Female, n (%)	91 (41.0)
Married or living with a partner, n (%)	198 (89.2)
Education, n (%)	
Have never gone to school	1 (0.5)
Grades 5th or less	3 (1.4)
9th to 12th grade, no diploma	44 (19.8)
High school graduate or GED completed	103 (46.4)
Some college level/ Technical/ Vocational degree	58 (26.1)
Bachelor’s degree	13 (5.9)
Advanced degree – Master’s and doctoral degree	0 (0)
Race, n (%)	
Black	166 (74.8)
White	37 (16.6)
Other (Asian, Native American, and Latin/Hispanic American)	19 (8.6)
Income, n (%)	
Less than $15,000	145 (65.3)
$15,000 - $19,999	22 (9.9)
$20,000 - $24,999	16 (7.2)
$25,000 - $34,999	23 (10.4)
$35,000 - $49,999	11 (5.0)
$50,000 - $74,999	2 (0.9)
$75,000 - $99,999	3 (1.4)
Subjective health, M (SD)	2.90 (1.04)
Self-efficacy, M (SD)	3.05 (0.62)
Emotional/informational social support, M (SD)	3.60 (1.04)
Tangible social support, M (SD)	3.41 (1.28)
Affectionate social support, M (SD)	3.70 (1.20)
Positive social interaction social support, M (SD)	3.62 (1.20)
Hopefulness, M (SD)	2.21 (1.17)

The multivariate regression analyses are presented in Table 2. After adjusting for covariate variables involving age, marital status, gender, education levels, race, and subjective health, those with higher self-efficacy were likely to report higher hopefulness (b = 0.60, se = 0.13, *p* < 0.001). Regarding social support, all subscales, such as emotional/informational (b = 0.24, se = 0.8 *p* = 0.003), tangible (b = 0.17, se = 0.6, *p* = 0.008), affectionate (b = 0.19, se = 0.7, *p* = 0.004), and positive social interaction social support (b = 0.17, se = 0.07, *p* = 0.012), were associated with higher rates of hopefulness.

**Table 2:** Multivariate regression analyses for predicting hopefulness

	b	se
Self-efficacy	0.60***	0.13
Emotional/informational social support	0.24**	0.08
Tangible social support	0.17**	0.06
Affectionate social support	0.19**	0.07
Positive social interaction social support	0.17*	0.07

Adjusted for age, marital status, gender, education levels, race, and subjective health; *p < 0.05; **p < 0.01; ***p < 0.001

**Fig. 1:**
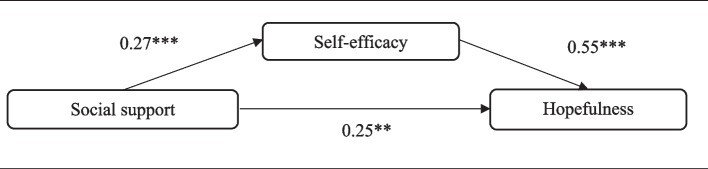
Mediation model of self-efficacy in the relationship between social support and hopefulness

Mediation analysis was conducted to assess the mediating role of self-efficacy in the relationship between social support and hopefulness in Fig. 1. Our mediation analysis revealed a partial mediation, which predicted not only a direct effect from self-esteem to hopefulness but also direct and indirect effects from social support to hopefulness. The direct effect of social support on self-efficacy (b = 0.27, se = 0.04, *p* < 0.001) and hopefulness (b = 0.25, se = 0.08, *p* = 0.001) was positive and significant. In addition, the direct association between self-efficacy and hopefulness in the mediation effect was positive and significant (b = 0.55, se = 0.14, *p* < 0.001). Finally, the indirect effect of social support via self-efficacy was positive and statistically significant (Effect = 0.15, Bootse = 0.04, BootLLCI - BootULCI = 0.07 - 0.25).

## Discussion

This study examined the effect of social support and self-efficacy on hopefulness among a sample of predominantly Black older adults living in a marginalized community during the COVID-19 pandemic. This present study is unique in determining the predictors that improve hopefulness in marginalized older adults during the pandemic. Our results support the hypothesis that social support and self-efficacy are significant predictors of hopefulness.

Higher self-efficacy and social support subscales were associated with higher hopefulness in our sample. Regarding mediation analysis, the direct and indirect effect of social support via self-efficacy was positively associated with hopefulness, which is partial mediation. These findings are in line with those of previous studies, which found that perceived social support [51–53] and self-efficacy [54, 55] were more likely to improve mental health among older adults. Despite the abundant studies on the effects of social support and self-efficacy on adverse mental health, to our best knowledge, there is a lack of studies focusing on hopefulness among marginalized older Black adults during the pandemic. Previous studies also indicated that older adults with higher hopefulness were likely to have higher life satisfaction [56] and lower mortality [57]. Given these beneficial effects of hopefulness on health outcomes, our study further extends the previous studies by focusing on positive psychological well-being in marginalized older adults during the pandemic.

Marginalized older adults tended to be self-isolated to prevent the COVID-19 virus infection, worsening their pre-existing social isolation [58]. Older Americans showed higher levels of social support (MOS-SSS: 4.05) than participants in our study (MOS-SSS: 3.58). In addition, older White Americans with high education levels (i.e., Bachelor’s, Master’s, and Doctorate degrees) received more tangible support (i.e., tasks and goods) during the pandemic than the pre-pandemic era [59]. Specifically, our participants showed lower levels of tangible social support (M = 3.41) than general older Americans from an existing study (M = 3.98) [60]. The low levels of social support could increase adverse mental health (i.e., loneliness) among older adults during the pandemic [58].

Findings from the partial mediation model in our study raise intriguing questions about how social support from spouses, family members, and friends and social engagement could work as a protective factor to increase positive psychological well-being among marginalized older adults. Our study focused on four factors of social support such as emotional-informational, tangible, affectionate, and social interaction supports. However, social support patterns and processes within multigenerational households can be negatively and positively changed during the COVID-19 pandemic. For example, Black middle-aged adults provided more support to their older adults in households than children or younger adults but fewer support resources than White counterparts [22]. In addition, strong social support could foster collective solidarity and resilience as potential mediators, which improve the quality of life in older adults [61]. Thus, future research is required to investigate the potential buffering factors that enhance psychological well-being in older adults.

Advanced technologies can boost social support and participation in marginalized groups, such as ethnic minorities or persons with low-income. The restrictions in access to healthcare and social service facilities were likely to aggravate maintaining a healthy life during the pandemic [32]. Prior studies have illustrated that lower social support and participation were significantly associated with increased incidents of mental health disorders [54, 62], which was more critical to marginalized groups during the COVID-19 pandemic. With this concern in mind, advanced technologies (i.e., mobile, tablet, computer, and virtual reality) can enable them to alleviate social isolation via virtual social participation [63–65]. However, there exist barriers to access (e.g., cost and support), feasibility (e.g., perceived safety), and motivation to use the technologies in marginalized older adults [63]. Considering these barriers and diverse preferences, further studies need to discover innovative methods to motivate marginalized older adults to participate in social activities using advanced technologies.

These findings underscore the importance of improvements in social support and self-efficacy when designing and implementing positive psychological interventions to enhance hopefulness among older adults. Our findings support a previous study on identifying the effects of social support and self-efficacy on boosting positive emotions [66]. Previous clinical trials found that positive psychological interventions (e.g., loving-kindness meditation or happiness, goal setting, and resource-building assignments) could train healthy adults to enhance personal resources (e.g., resilience and social support) and self-efficacy, which possibly improve hopefulness [67, 68]. These findings suggest that positive psychological interventions aimed at promoting hopefulness in older adults should prioritize strengthening social support and self-efficacy.

Despite these significant implications of our study, there are several limitations. Our study involved small sample sizes. According to power calculation, considering the estimated size of power values (> 0.8) for mediation analysis, the sample size should be more than 462 participants [69]. Even though we implemented 5,000 bootstrap resampling techniques using the PROCESS macro version 4.2, it remains a minor limitation of our study. Furthermore, as for the specific demographic characteristics in our study, it is not known whether these results would be generalizable to older adults of other ethnicities or residing in different locations. In addition, as a cross-sectional study, our findings cannot develop the temporal associations between the exposure (i.e., social support and self-efficacy) and the outcomes (i.e., hopefulness). Further study needs to focus more on identifying the relationships via a longitudinal database and adequate sample size.

## Conclusion

This current study found that more social support predicted greater hopefulness, and self-efficacy worked as a mediator in the relationship among marginalized older adults aged 50 and over. These findings emphasize the role of social support and self-esteem in improving positive psychological well-being. Given our findings, we can provide innovative and appropriate interventions to improve mental health for marginalized older adults. Future study is possibly strengthened using a longitudinal dataset and a larger sample size.

## Data Availability

The datasets used and/or analyzed during the current study are available from the PIs (Liliane Windsor and Ellen Benoit) upon reasonable request.

## Abbreviations

COVID-19: Coronavirus disease 2019
CBPR: Community-based participatory research
CCB: Community collaborative board
GSE: General Self-Efficacy Scale
MOS-SSS: MOS Social Support Survey
